# Endothelial function is impaired in conduit arteries of pannexin1 knockout mice

**DOI:** 10.1186/1745-6150-9-8

**Published:** 2014-05-17

**Authors:** Dina Gaynullina, Olga S Tarasova, Oxana O Kiryukhina, Valery I Shestopalov, Yuri Panchin

**Affiliations:** 1 Department of Human and Animal Physiology, Faculty of Biology, M.V. Lomonosov Moscow State University, Leninskie Gory 1-12, 119234 Moscow, Russia; 2 Department of Physiology, Russian National Research Medical University, Ostrovityanova str. 1, 117997 Moscow, Russia; 3 State Research Center of the Russian Federation – Institute for Biomedical Problems RAS, Khoroshevskoe shosse 76A, 123007 Moscow, Russia; 4 Department of Ophthalmology, Bascom Palmer Eye Institute, University of Miami, Miller School of Medicine, Miami, Florida, USA; 5 Vavilov Institute of General Genetics, Russian Academy of Sciences, Moscow, Russian Federation; 6 Institute for Information Transmission Problems, Russian Academy of Sciences, Bolshoi Karetny pereulok 19-1, 127994 Moscow, Russia; 7 Department of Mathematical Methods in Biology, Belozersky Institute, M.V.Lomonosov Moscow State University, Leninskie Gory 1-40, 119991 Moscow, Russia

**Keywords:** Pannexin1, Endothelium, Saphenous artery, Knockout mice

## Abstract

Pannexin1 is ubiquitously expressed in vertebrate tissues, but the role it plays in vascular tone regulation remains unclear. We found that Pannexin1 expression level is much higher in the endothelium relative to smooth muscle of saphenous artery. The ability of endothelium-intact arteries for dilation was significantly impaired whereas contractile responses were considerably increased in mice with genetic ablation of Pannexin1. No such increased contractile responses were detected in the endothelium-denuded arteries. Combined, our findings suggest a new function of Pannexin1 as an important player in normal endothelium-dependent regulation of arterial tone, where it facilitates vessel dilation and attenuates constriction.

Reviewed by Dr. Armen Mulkidjanian and Dr. Alexander Lobkovsky.

## Findings

### Introduction

Cell-to-cell communication provides coordination of cellular processes in multicellular organisms. In the vascular system, multilayer communication takes place between cells of different types [1]. Known mechanisms underlying this essential communication include gap junctions (GJ) [1,2] and paracrine action of signaling molecules, such as ATP [3]. GJ are composed of the connexin family of proteins that connect the cytoplasm of adjacent cells by forming a transmembrane channel permeable to ions and small molecules. Connexins, identified as the molecular components of GJ almost 30 years ago [2], were only found in chordates [4,5]. Different family of functionally similar but structurally unrelated GJ proteins was found in invertebrates. This family of specific invertebrate GJ proteins was originally designated OPUS [6] and, later renamed into innexins [7]. After innexin homologs were discovered in Humans and other vertebrates, it was proposed to reclassify them along with their vertebrate homologs into a bigger family, named pannexins [8,9]. Mammalian pannexins has three family members (Panx1, 2 and 3) that are involved in GJ formation [10,12] and/or form hemichannels implicated in the release of ATP and other small molecules from the cytoplasm into extracellular milieu. Some investigators claim that vertebrate pannexins form only hemichannels in vivo [13,14]. Since pannexins are involved in intercellular communication that is essential for the functioning of the vascular system [1], the presence (or absence) of pannexins will affect blood flow regulation.

Two major functional components of vascular wall are smooth muscle and endothelium. The endothelium mediates vasodilator effects of many blood-circulating hormones and locally acting autacoids [15]. In addition, the endothelium may tonically suppress vessel constriction even without direct activation; the phenomenon assigned as an anticontractile effect of the endothelium [16,17]. Noteworthy, the impaired endothelial function in both resistance-size and larger conduit arteries is considered a major cause of many cardiovascular disorders [18].

In murine systemic arterial network Panx1 is the primary expressed isoform [19]. Panx1 is abundant in endothelium of all arteries [19] and capillaries [5], but the pattern of Panx1 expression depends on the vessel size. In contrast to larger conduit arteries where Panx1 is expressed primarily in the endothelium, in smaller resistance arteries it is also expressed in smooth muscle cells [19]. It has been demonstrated that Panx1 is the essential pathway for ATP release that potentiates skeletal muscle contraction [20]. By similarity, in the smooth muscle of small arteries, Panx1-mediated ATP release was shown to participate in vascular tone regulation by potentiating arterial contractile response to α_1_-adrenoceptor activation [21]. However, functional role of Panx1 in facilitating vasodilator and anticontractile effects of the endothelium has never been studied before.

This study, for the first time, presents evidences supporting a critical role of Panx1 in endothelium-dependent component of vessel dilation mechanism. Since truly selective pharmacological blockers of the Panx1 channel currently are not available, we utilized Panx1^−/−^ mice as the ultimate test model for investigating functional significance of Panx1 activity in vascular system regulation.

## Results and discussion

**Figure 1:**
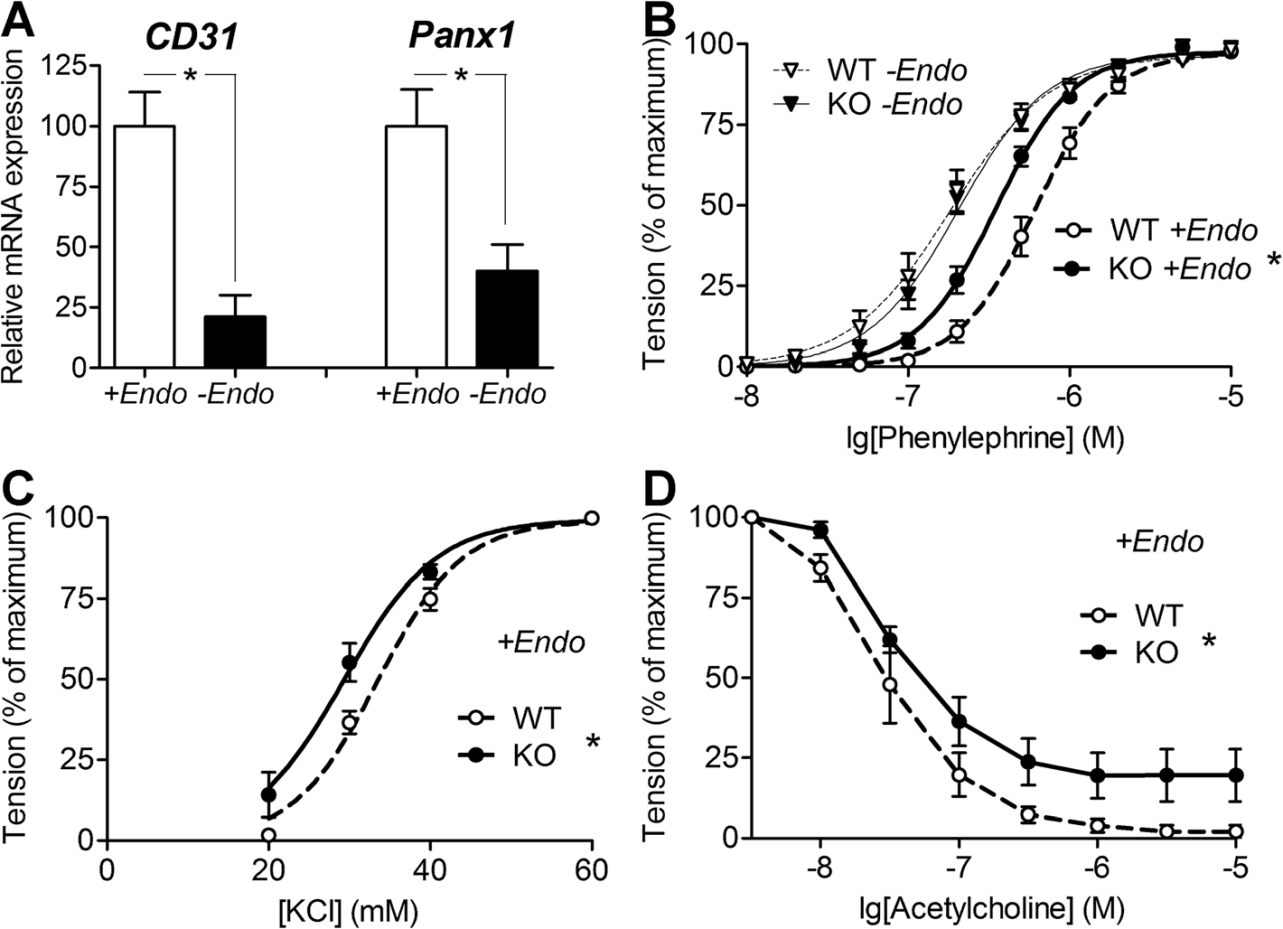
**Endothelial function is impaired in saphenous arteries of Panx1**^**−/−**^**mice. (A)** Relative CD31/SM22 and Panx1/SM22 mRNA expression levels in endothelium-intact (*+Endo*) and endothelium-denuded (−*Endo*) arteries of WT mice (n = 4; 4 for CD31 and n = 3; 4 for Panx1; *p < 0.05,). Data were normalized to the amount of smooth muscle cells marker SM22 mRNA and expressed as % of the average level in corresponding endothelium-intact (*+Endo*) group. **(B)** Concentration-response relationships to cumulative addition of α_1_-adrenoceptor agonist phenylephrine of *endothelium-denuded* (thin curves; n = 11, 10, p = 0.14) or *endothelium-intact* (thick curves; n = 22, 24, *p < 0.05) saphenous arteries from WT and Panx1^−/−^ mice. **(C)** Concentration-response relationships of *endothelium-intact* saphenous arteries from WT and Panx1^−/−^mice to KCl (n = 6; 9, *p < 0.05; the effect of noradrenaline released from sympathetic nerves was blocked by 1 μmol/L phentolamine). **(D)** Concentration-response relationships to acetylcholine after phenylephrine-induced preconstruction of *endothelium-intact* saphenous arteries from WT and Panx1^−/−^mice (n = 6, 6, *p < 0.05).

Lohman et al. [19] used immunohistochemistry to reveal primarily endothelial localization of Panx1 in murine systemic conduit arteries. However, due to poor specificity of most available antibodies against this protein [22], quantitative immunohistochemistry data for Panx1 remains rather unreliable. Here, we addressed this concern by alternative, more quantitative approach, i.e. by comparing Panx1 gene expression levels in endothelium-intact and endothelium-denuded arterial preparations (Figure 1A; Additional file 1: Figure S1). As expected, the disruption of the endothelium caused significant reduction of the transcript for endothelial marker CD31. Accordingly, the endothelium removal resulted in a considerable drop in the Panx1 transcript, thus indicating that Panx1 is expressed predominantly in endothelial cells of murine saphenous artery. In the saphenous arteries from Panx1^−/−^ mice, no Panx1 transcript has been detected by PCR amplification with the same primers.

Vascular tone in rodent saphenous artery is regulated mainly by sympathetic nerves that release noradrenaline, which causes vasoconstriction predominantly through α_1_-adrenoceptors. To mimic the constrictor effects of noradrenaline, we examined contractile responses to α_1_-adrenergic agonist phenylephrine and the regulatory role of endothelium in these contractions in WT and Panx1^−/−^ mice. The α_1_-adrenoceptors are located only on smooth muscle cells, not on the endothelium; thereby phenylephrine stimulates primarily smooth muscle. Phenylephrine-induced contractile responses of *endothelium-denuded* arteries did not differ in WT and Panx1^−/−^ mice (Figure 1B), which is in good agreement with predominantly endothelial localization of Panx1 in murine saphenous arteries (Figure 1A).

In WT mice, the *endothelium-intact* arteries were less sensitive to phenylephrine compared to endothelium-denuded arteries (Figure 1B) due to the anticontractile action of the endothelium [16,17]. Importantly, such influence of the endothelium in the arteries from Panx1^−/−^ was prominently weaker than that in WT mice. This is consistent with a model where Panx1 mediates and the ablation of Panx1 attenuates the anticontractile action of the endothelium. As Figure 1B shows, when phenylephrine was applied in concentration of 2*10^−7^ M (point −6.7) the responses in KO mice were more than two-fold stronger than in WT mice. Quantitatively, Panx1 activity can be accounted for approximately half of the anticontractile action of the endothelium.

Noteworthy, phenylephrine can be taken up by periarterial sympathetic nerves similar to a natural transmitter, noradrenaline. Therefore, the observed differences between WT and Panx1^−/−^mice (Figure 1B) might be explained by altered neuronal uptake, as was demonstrated in vessel denervation model [23]. However, the enhancement of contractile responses in *endothelium-intact* arteries in Panx1^−/−^ vs. WT mice was also observed during application of methoxamine (Additional file 2: Figure S2), which activates α_1_-adrenoceptors but is not taken up by sympathetic nerves [24]. Similar enhancement was observed when the contractile response was induced by non-receptor activation by high-K^+^ depolarization (Figure 1C). This demonstrates that anticontractile effect of Panx1 is not restricted to α_1_-adrenergic agonists and may appear in response to a variety of stimuli.

Another strong evidence for functional role of Panx1 in the endothelium was obtained by comparing the acetylcholine-induced relaxations of *endothelium-intact* arteries in WT and Panx1^−/−^ mice. Acetylcholine affects arterial tone exclusively though receptors on endothelial cells, therefore the endothelium-denuded arteries do not dilate to acetylcholine (data not shown). We found that the response to acetylcholine was significantly weaker in Panx1^−/−^ vs. WT saphenous arteries (Figure 1D), indicating that Panx1 participates in the regulation of the endothelium-dependent dilatory mechanisms.

Three putative Panx1-mediated pathways in the vascular endothelium are worth further exploration: either Panx1-formed gap junction channels interconnecting endothelial cells, as suggested previously [5] or via Panx1 hemichannel-mediated Ca^2+^-influx, and/or ATP release [25,26]. Extracellular ATP may induce relaxation either itself, via endothelial P2 receptors [3], or after degradation by ectonucleotidases to adenosine, a potent vascular dilator [3,27]. Our findings of the decreased endothelium-dependent relaxation and the anticontractile action of the endothelium in Panx1^−/−^ mice suggest the participation of Panx1 in these mechanisms.

In conclusion, our data demonstrate for the first time that Panx1 is involved in the regulation of endothelium-dependent relaxation of conduit arteries. We show that Panx1 mediates endothelial contribution to the adrenergic-induced contractions, thereby modulating vascular tone. The results of this study suggest that activity of Panx1 in the endothelium of conduit arteries is important for control of blood supply to different organs. Activation of the endothelium in conduit arteries during episodes of mechanical stimulation (shear stress) by increased blood flow ensures their dilation and thereby stabilizes pressure drop along the proximal site of vascular bed [28]. Therefore, the blockade or ablation of Panx1 in the endothelium may disturb a proper adjustment of proximal and distal vascular resistances, a necessary condition for adequate control of regional blood flow and maintaining the level of arterial blood pressure.

## Methods

### Animals

Panx1^−/−^ mouse strain was described previously [29]. All experiments in this study were performed in full compliance with the NIH Guide for the Care and Use of Laboratory Animals and Russian national guidelines for animal research. The protocols were approved by University of Miami IACUC (protocol #12-051) and Institute for Information Transmission Problems, Russian Academy of Sciences IACUC (protocol #02-2013). Wild type (WT) animals were age-matched (2–3 months old) male mice of the C57BL/6 background. Mice were housed under standard conditions of temperature and humidity, with a 12-hour light/dark cycle and free access to food and water. Mean body weights: WT – 28.9 ± 0.6 g, Panx1^−/−^ - 26.2 ± 0.4 g (p < 0.05).

The experiments were performed using the preparations of the saphenous artery, which branches off from the femoral artery at the level of the knee and supplies blood to the foot [30]. The inner diameters of saphenous arteries in WT and Panx1^−/−^mice were 272.4 ± 5.5 micron and 304.9 ± 5.8 micron respectively. For force recording, 2-mm ring preparations were mounted in isometric myograph [31] (for details see Additional file 3). Standard methods were used for gene expression and statistical analysis (see Additional file 3).

## Reviewers’ comments

### Referee 1 - Dr Armen Mulkidjanian

“Biology Direct” aims on a broad audience of biologists, therefore the manuscript should be amended to bring the finding of the authors in a broader biological context. Accordingly, the Introduction and Discussion sections should be expanded.

### Author response

Dear reviewer, thank you very much for your reviewing on our manuscript. We expanded the Introduction section (within the text size limitations for Discovery notes) to address it to a broader audience. As well, we supplemented the Discussion by phrases on functional consequences of Panx1 deficiency.

### Referee 2 - Dr Alexander Lobkovsky

The article presents measurements of contractile response of mammalian (mouse) arteries to several stimuli in mice with Panx1 knockout and/or endothelium denuded blood vessel. The conclusion namely that Panx1 is involved regulation of endothelium dependent relaxation of blood vessels is well supported and clearly argued. Methods (both in the lab and data analysis) are clearly stated and whenever statistical significance is claimed, appropriate p-values are computed.

## Competing interests

The authors declare that they have no competing interests.

## Authors’ contributions

OST and YP planned the project; DG, OST, OOK, VIS and YP performed experiments, data evaluation, interpretation and manuscript preparation. VIS generated the mouse model. All authors read and approved the final manuscript.

## Supplementary Material

Additional file 1: Figure S1 RT-PCR products for smooth muscle cells marker SM22, endothelial cell marker CD31 and Panx1 in endothelium-intact (*+Endo*) and endothelium-denuded (−*Endo*) arteries. Click here for file

Additional file 2: Figure S2 Concentration-response relationships of *endothelium-intact* saphenous arteries from WT and Panx1^−/−^ mice to methoxamine (n = 7; 10, *p < 0.05). Click here for file

Additional file 3 Methods. Click here for file
