# Supplementary material for: Endothelial function is impaired in conduit arteries of pannexin1 knockout mice
Source: Biol Direct. 2014 May 17;9:8. doi: 10.1186/1745-6150-9-8 (PMC4046076; doi:10.1186/1745-6150-9-8)
Supplement: Additional file 1: Figure S1 — RT-PCR products for smooth muscle cells marker SM22, endothelial cell marker CD31 and Panx1 in endothelium-intact (+Endo) and endothelium-denuded (−Endo) arteries. [file 1745-6150-9-8-S1.pdf]

Additional file 1: Figure S1.

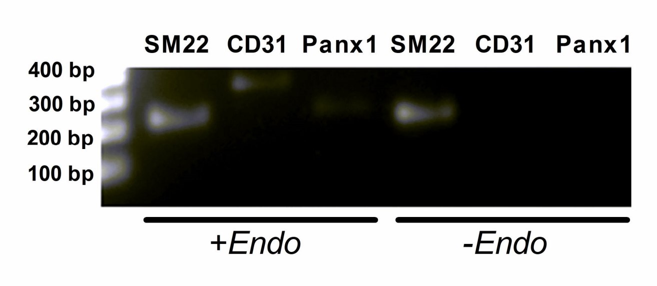


RT-PCR products for smooth muscle cells marker SM22, endothelial cell marker CD31 and Panx1 in endothelium-intact (+*Endo*) and endothelium-denuded (-*Endo*) arteries.
