# Supplementary material for: Endothelial function is impaired in conduit arteries of pannexin1 knockout mice
Source: Biol Direct. 2014 May 17;9:8. doi: 10.1186/1745-6150-9-8 (PMC4046076; doi:10.1186/1745-6150-9-8)
Supplement: Additional file 2: Figure S2 — Concentration-response relationships of endothelium-intact saphenous arteries from WT and Panx1−/− mice to methoxamine (n = 7; 10, *p < 0.05). [file 1745-6150-9-8-S2.pdf]

Additional file 2: Figure S2.

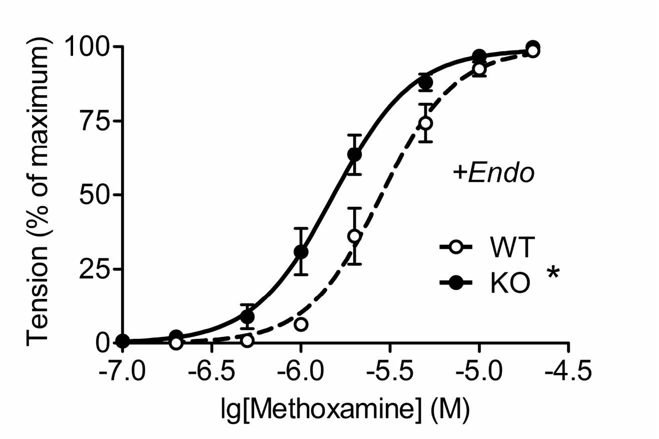

Concentration-response relationships of *endothelium-intact* saphenous arteries from WT and Panx1$^{-/-}$ mice to methoxamine (n=7; 10, *p<0.05).
