# Supplementary material for: Endothelial function is impaired in conduit arteries of pannexin1 knockout mice
Source: Biol Direct. 2014 May 17;9:8. doi: 10.1186/1745-6150-9-8 (PMC4046076; doi:10.1186/1745-6150-9-8)
Supplement: Additional file 3 — Methods. [file 1745-6150-9-8-S3.pdf]

Additional file 3. Methods.

Gene expression

Gene expression levels were measured by quantitative PCR, performed in Rotor Gene 6000 (Corbett Research, Australia) with SYBR Green master mix (Syntol, Russia) and the following protocol: 95 °C for 10 min, followed by 40 cycles, consisting of three phases: 30 s at 95 °C, 30 s at 66°C and 60 s at 72°C. Primer sequences (5'-3') for SM22: forward CACAAACGACCAAGCCTTCTC, reverse TCACCAATTTGCTCAGAATCACA; for CD31: forward GGACTCACGCTGGTGCTCTATGC, reverse CACCTTGGGCTTGGATACGCCATG; for Panx1: forward CTCCCACTGTGGCTGCACAAGTT, reverse AGATATCTCCCACAGACTCTG. To avoid possible amplification from genomic DNA, all primers were designed to span intron and to continue to the adjacent exon by 3 nucleotides on the 3' prime end.

Wire myography

Saphenous arteries were isolated and mounted in a wire myograph (DMT, Denmark, Model 620M) for the recording of isometric force. Physiological salt solution contained (in mM): 120 NaCl, 26 NaHCO, 4.5 KCl, 1.6 $CaCl_2$, 1 $MgSO_4$, 1.2 NaH_2PO_4, 5.5 D-glucose, 0.025 EDTA and 5 HEPES with pH 7.4 was used throughout the experiment. To obtain endothelium-denuded arteries, we used small rat's whisker for endothelium disruption. The vessels were stretched to an internal circumference at which they developed maximal active tension [23] and kept at 37°C, bubbled with a gas mixture (95% O_2, 5% CO_2) for maintaining pH at 7.4. Arteries were activated 2 times with 10 µmol/L phenylephrine and the functional activity of the endothelium was checked subsequently using 10 µmol/L acetylcholine during 1-3 µmol/L phenylephrine-induced preconstriction. Contractile responses were studied during cumulative addition of vasoactive agonists. Isotonic high-KCl solutions were prepared by equimolar substitution of NaCl to KCl.

Statistical analysis

All data are expressed as mean ± SEM; n represents the number of animals tested. Concentration-response relationships were evaluated by using Repeated Measures ANOVA to detect differences between groups (WT vs. KO). Statistical significances for relative mRNA expression were determined using unpaired Student t-test. Statistical significance was reached at $p<0.05$.
